# Cystic Echinococcosis/Hydatid Cyst Coinfection with HIV: A Report from Shiraz, Iran

**DOI:** 10.1155/2021/8844104

**Published:** 2021-02-20

**Authors:** Yosef Sharifi, Seyed Mahmoud Sadjjadi, Hamed Nikoupour Dailami, Seyed Hamed Jafari, Mohammad Hossein Anbardar, Mohammad Bagher Khosravi

**Affiliations:** ^1^Department of Parasitology and Mycology, School of Medicine Shiraz University of Medical Sciences, Shiraz, Iran; ^2^Basic Sciences in Infectious Diseases Research Center, Shiraz University of Medical Sciences, Shiraz, Iran; ^3^Department of Transplantation, Shiraz University of Medical Sciences, Shiraz, Iran; ^4^Medical Imaging Research Center, Shiraz University of Medical Sciences, Shiraz, Iran; ^5^Department of Pathology, Shiraz Transplant Center, Abu Ali Sina Hospital, Shiraz University of Medical Sciences, Shiraz, Iran; ^6^Department of Anesthesia, Shiraz Anesthesia and Critical Care Research Center, Shiraz, Iran

## Abstract

HIV coinfected with other parasitic diseases may cause a serious problem for the patients. A few case reports describing echinococcosis with human immunodeficiency virus (HIV) infection have been reported in the world; however, it has not been reported in Iran, so far. Here, the first case of liver hydatid cyst coinfected with HIV in Iran is reported. The patient is a 46-year-old female HIV-positive based on the laboratory report. Her clinical symptoms included abdominal pain, abdominal enlargement, and anorexia. Ultrasound showed three large hepatic hydatid cysts with hundreds of daughter cysts. Ultrasonography of the cyst revealed it as a CE2 stage according to the WHO classification. The patient went under complete anesthesia followed by complete cyst removal by surgery. Observation of the hydatid cyst fluid using eosin 0.1% revealed more than 70% viable protoscoleces. Histopathology examination, polymerase chain reaction (PCR), and viable protoscoleces confirmed the diagnosis of echinococcosis. The IgG ELISA test with native AgB for *E. granulosus* infection was also positive. mtDNA amplification using PCR and sequencing showed the cyst as *E. granulosus* sensu stricto genotype. Our observations show that huge, large, and high-pressure cysts with hundreds of daughter cysts are difficult to be completely removed, and drug treatment has not been able to reduce their size. Therefore, in HIV coinfection with hydatid cyst, surgery is preferable to other treatments.

## 1. Introduction

Cystic echinococcosis (CE) is an important zoonotic parasite infection that is caused by the larval form of *Echinococcus granulosus* sensu lato (s.l.) [1]. *E. granulosus* s.l. consists of *E. granulosus* sensu stricto (s.s.) (genotypes G1–G3), *E. equinus* (genotype G4), *E. ortleppi* (genotype G5), *E. canadensis* cluster (genotypes G6–8 and G10), and *E. felidis* [2]. Human is a random intermediate host for the parasite whom the larval stage or metacestode can be located in different organs of this host [3]. The prevalence of CE in different organs reaches 70% in the liver, 20% in the lung, and 10% in other organs (brain, muscle, pericardium, kidney, eyes, and bone marrow) [4]. Diagnosis of hydatid cyst is based on clinical symptoms, imaging (ultrasonography, CT scan and MRI), and serologic methods, especially based on the use of native antigens B [5]. Ultrasonography is usually the first diagnostic line which is classified by WHO as CE1 to CE5 [6].

The disease has been reported in humans from all parts of Iran so that a total of 1% of patients admitted to the surgery departments harbored this disease [7]. Although parasitic infections are unusual by cestodes in HIV-infected patients [8], hydatid cyst has been reported in HIV patients in Australia, Turkey, India, Spain, Romania, Netherlands, Switzerland, and Brazil [9–19]. However, the hydatid cyst has not been reported in HIV patients in Iran, so far. This study aimed to report rare cases of hydatid cyst coinfected with HIV in Iran so that the cysts were significantly enlarged, and this may be due to a defective immune system. Surgery is very important in the recovery and survival of the patient.

## 2. Case Presentation

**Figure 1:**
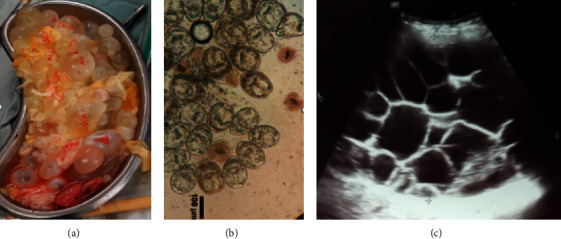
(a) Hundreds of daughter cysts removed from CE patient. (b) Using eosin 0.1% on the protoscoleces collected from cysts revealed more than 70% viability (viable protoscoleces do not stain by eosin). (c) Abdominal ultrasound showing a large multiloculated cyst on the liver which was classified as CE2 stage according to the WHO classification.

**Figure 2:**
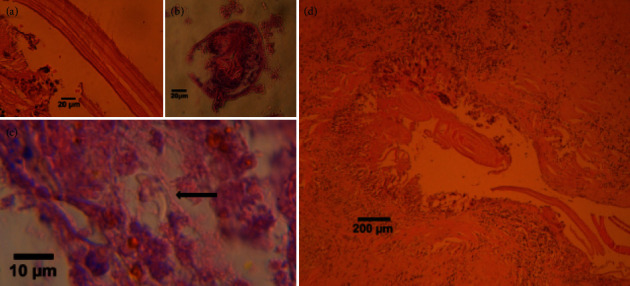
Section of hydatid cyst stained with H&E. (a) Laminated membrane. (b) Protoscolex (with hook and calcareous body). (c) Hooklet. (d) Destruction of liver parenchyma by the hydatid cyst and replacement by fibrosis and giant cell.

A 46-year-old female with HIV infection was admitted to Abu Ali Sina hospital, with some clinical symptoms including abdominal pain, abdominal enlargement, and anorexia. Symptoms of hydatid cyst started with abdominal pain about a year before surgery, and over time, as the abdomen enlarged, anorexia appeared. She is a housewife, living in the village (around Shiraz, Iran), and in contact with domestic animals (sheep, cows, goats, etc.) and dogs. She contracted HIV around 2014 from having sex with her husband and was treated for HIV immediately after diagnosis, and the HIV viral load (copies/ml) was not detected before surgery. Based on her clinical signs, abdominal ultrasound was done which showed three large multiloculated cysts on her liver with a size of 100 × 80 mm in the right lobe, 74 × 55 mm in the subdiaphragmatic aspect, and 200 × 130 mm adjacent to the lower border of the left lobe. The stage of all cysts was CE2 according to the WHO classification (Figure 1(c)). The IgG ELISA test using native AgB on the sera of the patient revealed a positive result for CE. The patient went under complete anesthesia, followed by partial cystectomy with the following procedure: incision and drainage of the cysts, closure of biliary fistulas, T-tube insertion in the common bile duct, and two mushroom drain insertion in cyst cavities. Hypertonic saline was used as a scolicidal agent during surgery. All three cysts possess hundreds of daughter cysts (Figure 1(a)). The patient was treated with albendazole (400 mg twice daily) for 4 weeks before surgery and the same dose for 3 months after surgery. The cysts were fertile so that using eosin 0.1% on the protoscoleces collected from cysts revealed more than 70% viability (Figure 1(b)). Histopathology of the specimens (Figure 2) showed the destruction of liver parenchyma by hydatid cyst and replacement by fibrosis and foreign body-type giant cell reaction (Figure 2(d)), and sections of a laminated membrane, a protoscolex, and a hooklet of CE (Figures 2(a)–2(c), respectively).

The laboratory data are in Table 1 which shows red blood cells (RBCs), hemoglobin (Hb), hematocrit (HCT) were less than normal pre- and postsurgery. Although MCV and MCH and alkaline phosphatase (ALP) were higher than normal. DNA was extracted from protoscoleces for PCR. Application of specific primers JB3 and JB4.5 for the cox1 gene [20] followed by sequencing showed the cyst as *E. granulosus* sensu stricto genotype (accession number: MT073987). The patient has a normal life 18 months after surgery.

## 3. Discussion

Cystic echinococcosis is widespread in many parts of the world including Mediterranean countries where many dogs and their environments are infected. So, the people living in these areas are at risk of infection. Humans are usually infected by ingesting the eggs of the parasite from the environment and dogs [21]. Hydatidosis is a silent disease, especially in the case of liver involvement. Therefore, the cysts may survive for a prolonged period and sometimes do not display outstanding symptoms. Clinical symptoms may develop when the cysts become large enough to press other organs and causing pain or cause visual abdominal swelling. Frequently, patients complain of abdominal pain in the right upper quadrant [22]. Abdominal pain has been the main symptom sometimes together with hepatomegaly, epigastric tenderness, jaundice, and abdominal enlargement [9, 10, 12, 13]. Our case had abdominal pain, abdominal enlargement which was common to other reported symptoms, and also anorexia which was not reported in other cases [9, 10, 12, 13].  Table 2 compares cases of coinfection with CE and HIV.

HIV coinfection with cestode diseases has been reported, including CE, alveolar echinococcosis (AE), polycystic echinococcosis, and neurocysticercosis [9–19], but HIV coinfection with CE is more common which could be due to the higher prevalence of *E. granulosus* in the world [9–19]. Such coinfected disease has been reported in hepatic CE, spinal CE, pulmonary CE, renal CE, and hepatorenal CE [9–19]. All previous liver cases were male, but our case was female. Although patients have been in the middle age (between 28 and 47 years), a 5-year-old boy with hepatorenal hydatidosis and AIDS also has been reported [9, 10, 12, 13].

Based on the WHO categorization of the stages of human liver CE [6], the isolated cyst was categorized as CE2 which is similar to three out of four patients identified in the work of Ran et al. [9]. Miron et al. showed presurgery chemotherapy of echinococcal infections can reduce the size and number of viable protoscoleces [23]. However, in our case, although she was treated with albendazole (400 mg twice daily) for 4 weeks before surgery, no change was observed in the size and number of live protoscoleces. In our case, the viability of protoscoleces was over 70%. Unfortunately, this has not been reported in other cases coinfected with CE [9, 10, 12, 13].

Both humoral and cellular immunity mechanisms play essential roles in response to human hydatidosis. Th1 and Th2 cell activation and the expression of immunoglobulin isotypes such as IgG and IgE occur at different stages of the infection [24, 25]. Current evidence about the level of IgG4 and IgE antibodies and frequent eosinophilia in hydatid disease suggests that the dominant immune response to *Echinococcus* infection is the response of the TH2 (T-helper) type [26]. In this case, eosinophilia was normal before surgery (1-2%) but increased significantly a few days after surgery (eosinophilia: 14%). This item has not been reported in other cases coinfected with CE [9, 10, 12, 13]. Hailong et al. have reported eosinophilia is not usually observed in patients with hydatid disease. In most cases, it is either mild (<15%) or absent; although in patients with ruptured cysts in the biliary tree, eosinophilia is often marked and shows transient elevation (up to 60%). In this study, eosinophil count was normal before the surgery, but it increased prominently after the surgery. A reason for this could be that some rupture occurred during surgical removal of the hydatid cysts [27]. Also, the increase in eosinophilia may be due to drug treatment 4 weeks before surgery, which is similar to the report of Di Comite et al. who stated eosinophilia increased from 30% to 48% after treatment [28].

HIV attacks to TCD4 cells and reduces them. Zingg et al. have suggested that especially CD4+ Th cell counts have been associated with the immune response to the parasite and the severity of *Echinococcus* infection [19]. The CD4 level of the present case was lower than normal, but not too low which could be due to AIDS medication. There are a few reports of CE-HIV patients with negative serology [9]. It could be because of the reduced immune response in HIV patients or due to differences in species or strain of parasite, location of the cyst, number of the cysts, cyst fertility, cyst viability, and the integrity of the cyst walls [29, 30].

Hydatid cyst growth is slow. Moro et al. reported that the rate of natural growth from 0.4 to 1.4 cm over 3 years [31]. HIV infection may aggravate hydatid cyst or accelerate cyst growth, producing huge or large cyst with even hundreds of daughter cysts [9, 18]. Chauchet et al. reported that, in patients infected with *E. multilocularis* and immunodeficiency, such as AIDS, the protoscoleces proliferation appear uncontrolled, leading to a very rapidly progressing disease status [32]. Finding the large cysts in the present patient could be due to HIV infection. A literature review (Table 2) showed that, from seven cases of CE-with HIV coinfection, a total of five cases have been gone under surgery or PAIR (percutaneous aspiration, injection, and reaspiration) [9, 10, 13], but in two cases who had refused surgery, death had occurred [9, 12]. The present case was also recovered after surgery.

## 4. Conclusion

Our observations showed while drug treatment has not been able to reduce the size of large cysts, complete removal of huge cysts with high pressure and hundreds of daughter cysts is also difficult. However, surgery is preferable to other treatments in cystic echinococcosis/hydatid cyst coinfection with HIV.

## Figures and Tables

**Table 1:** Comparison of the laboratory data including blood and sera tests, during pre- and postsurgery times shows the differences.

Laboratory test	Presurgery		Surgery	Postsurgery
	April 14, 2019	April 25, 2019	May 20, 2019	May 24, 2019
WBC (10^6^/L)	7.2	10.5		**L** ^*∗*^4.3
EOS (%)	2	1		**H** ^*∗*^14
RBC (10^6^/*µ*L)	2.39	2.15		2.3
Hb (g/dL)	**L** 9.3	**L** 8.3		**L** 8.2
HCT (%)	**L** 27.1	**L** 24.7		**L** 24
MCV (fL)	**H** 113.4	**H** 114.88		**H** 104.35
MCH (pg)	**H** 38.91	**H** 38.6		**H** 35.65
MCHC (g/dL)	34.32	33.6		34.17
Plt (10^6^/L)	281	414		297
BUN (mg/dL)	10	12		**L** 5
Cr (mg/dL)	0.85	1.0		0.7
AST (U/L)	22	18		15
ALT (U/L)	17	8		10
ALP (U/L)	230	**H** 352		**H** 314
Total bilirubin (mg/dL)	0. 58	0.69		0.43
Direct bilirubin (mg/dL)	0.27	0.29		0.23
Albumin (g/dL)	4.4	3.7		**L** 2.9
Total protein (g/dL)		8.8		6.6
CD4 T-cell count (420–1250 cells/ml)	**L** 377			**L** 333

^∗^ **L**: light; **H**: high.

**Table 2:** Comparison of cases of coinfection of CE with HIV.

	This study	Case 1 [9]	Case 2 [9]	Case 3 [9]	Case 4 [9]	Case 5 [13]	Case 6 [12]	Case 7 [10]
Location	Liver	Liver	Liver	Liver	Liver	Liver	Liver	Liver, kidney
Country	Iran	Australia	Australia	Australia	Australia	India	Spain	Romania
Age (year)	46	47	44	41	30	28	38	5
Sex	Female	Male	Male	Male	Male	Male	Male	Boy
Occupation	Housewife	Businessman	Unemployed	Farmer	Unemployed			
ELISA-based HIV	Positive	Positive	Positive	Positive	Positive	Positive	Positive	Positive
Serology for CE	Positive	Negative	Positive	Negative	Negative	Positive	Positive	Positive
Symptoms	Abdominal pain, abdominal enlargement, anorexia	Abdominal pain, fever, jaundice, epigastric tenderness	Pain, jaundice	Abdominal pain, epigastric tenderness, inguinal lymphadenopathy	Abdominal pain, tenderness	Abdominal pain, watery diarrhea, hepatomegaly, splenomegaly	Vomiting, cough, weight loss	Mild abdominal pain
Stage of cyst	CE2	CE2	CE2	CE3	CE2			
CD4 T-Cell count (cells/ml)	377–333	36.6	473	290		375	198	
Treatment for CE	Albendazole, refuse surgery	Pericystectomy, albendazole	Pericystectomy, albendazole	Pericystectomy, albendazole	Pericystectomy, albendazole	Laparoscopy, albendazole	Refuse surgery	Albendazole, PAIR^∗^
Other disease							HCV+	Giardiasis

^∗^PAIR: puncture-aspiration-injection-reaspiration.

## Data Availability

The data used to support the findings of this study are included within the article.
